# External Validation and Update of a Prediction Rule for the Duration of Sickness Absence Due to Common Mental Disorders

**DOI:** 10.1007/s10926-016-9646-1

**Published:** 2016-06-03

**Authors:** Giny Norder, Corné A. M. Roelen, Jac J. L. van der Klink, Ute Bültmann, J. K. Sluiter, K. Nieuwenhuijsen

**Affiliations:** 1 ArboNed Occupational Health Service, PO Box 85091, 3508 AB Utrecht, The Netherlands; 2 Department of Health Sciences, University Medical Center Groningen, University of Groningen, Groningen, The Netherlands; 3 School of Social and Behavioral Sciences, Tilburg University, Tilburg, The Netherlands; 4 Coronel Institute of Occupational Health, Academic Medical Center, University of Amsterdam, Amsterdam, The Netherlands

**Keywords:** Mental disorders, Prognosis, Return to work, Sick leave, Validation studies

## Abstract

*Purpose* The objective of the present study was to validate an existing prediction rule (including age, education, depressive/anxiety symptoms, and recovery expectations) for predictions of the duration of sickness absence due to common mental disorders (CMDs) and investigate the added value of work-related factors. *Methods* A prospective cohort study including 596 employees who reported sick with CMDs in the period from September 2013 to April 2014. Work-related factors were measured at baseline with the Questionnaire on the Experience and Evaluation of Work. During 1-year follow-up, sickness absence data were retrieved from an occupational health register. The outcome variables of the study were sickness absence (no = 0, yes = 1) at 3 and 6 months after reporting sick with CMDs. Discrimination between workers with and without sickness absence was investigated at 3 and 6 months with the area under the receiver operating characteristic curve (AUC). *Results* A total of 220 (37 %) employees agreed to participate and 211 (35 %) had complete data for analysis. Discrimination was poor with AUC = 0.69 and AUC = 0.55 at 3 and 6 months, respectively. When ‘variety in work’ was added as predictor variable, discrimination between employees with and without CMD sickness absence improved to AUC = 0.74 (at 3 months) and AUC = 0.62 (at 6 months). *Conclusions* The original prediction rule poorly predicted CMD sickness absence duration. After adding ‘variety in work’, the prediction rule discriminated between employees with and without CMD sickness absence 3 months after reporting sick. This new prediction rule remains to be validated in other populations.

## Introduction

Common mental disorders (CMDs) are an increasing burden of disease in the working population and a major cause of long-term sickness absence and disability pensioning [1–7]. In a systematic review, Blank et al. [8] found that only 50 % of the employees absent from work due to CMDs for 6 months or longer returned to work. The other half fails to resume work and ends up receiving a disability pension. If health care providers could use prognostic models and rules to identify employees at risk of long-term CMD sickness absence, then high-risk employees could be referred to treatment or targeted interventions soon after reporting sick. Such a tertiary preventive approach might improve the return to work prognosis.

Earlier, Nieuwenhuijsen et al. (2006) developed a prediction rule for CMD sickness absence duration. Employees aged >50 years, with a high educational level, who expected to be off work >3 months, and presented with depressive and/or anxiety symptoms were at risk of longer duration CMD sickness absence. Receiver Operating Characteristic (ROC) analysis showed that the prediction rule poorly discriminated between employees with and without sickness absence 3 months after reporting sick, with area under the ROC-curve (AUC) 0.68; discrimination was fair for employees with and without sickness absence 6 and 12 months after reporting sick, with AUC 0.71 and 0.73, respectively [9].

The prediction rule was developed in a rather homogeneous sample of 188 Dutch employees (54 % teachers). Prediction rules are practically useful only if they provide accurate risk predictions in different settings. The more heterogeneous the workplace settings in which the prediction rule is tested and found accurate, the more likely it will apply to untested settings [10]. Therefore, the aim of this study was to validate the rule predicting CMD sickness absence duration in a heterogeneous working population. In addition, we investigated the added value of work-related predictor variables to the original prediction rule for CMD sickness absence duration.

## Methods

### Study Design and Sample Size

The study was designed as a cohort study including employees working in companies with a sickness absence insurance and who reported sick with CMDs in the period September 2013 to April 2014. Predictor variables and work-related factors were measured at inclusion. Sickness absence data were retrieved from an occupational health register during 1-year follow-up. The Medical Ethics Committee of the University Medical Center Groningen approved the study (reference METc2011.204).

To calculate the sample size, we used a conservative estimate of 15 outcome events per variable as criterion [11]. As the prediction rule included 4 variables, we needed 60 employees still absent from work at 3, 6, and 12 months after reporting sick. A Dutch study showed that 53 % of the employees who reported sick with CMDs were still sick-listed at 3 months, 30 % at 6 months, and 13 % at 12 months after reporting sick, respectively [12]. Based on these percentages, we estimated that N = 100, N = 200 and N = 450 would have to be included to validate the prediction rule for CMD sickness absence at 3, 6, and 12 months, respectively.

### Data Collection

Employees who reported mental problems as cause of sickness absence in the period September 2013 to April 2014 were invited by e-mail to participate in the study. Those who agreed to participate received an online questionnaire measuring the established predictor variables and work-related factors. In The Netherlands, sickness absence is compensated when medically certified by an occupational physician (OP). OPs certify sickness absence with a diagnostic code based on the 10th version of the International Classification of Diseases (ICD-10). Of the employees who reported sick with mental problems, only those suffering CMDs were included in the analyses. CMD sickness absence was defined as OP-certified within ICD-10 diagnostic categories R45 (emotional disturbances), F30-39 (mood disorders), or F40-49 (neurotic disorders). Employees with other mental problems, OP-certified as schizophrenia (F20-29), personality disorders (F60-69), mental retardation (F70-79), and disorders of psychological development (F80-89) were excluded from the analyses, because these diagnoses were not considered CMDs [9]. Employees who were unable to understand or complete an online Dutch questionnaire were also excluded from the analyses.

### Outcome Variable

CMD sickness absence was recorded in an occupational health service register from the day of reporting sick to the day of full return to work (i.e. working the same number of hours per week as before CMD sickness absence). Based on the duration of CMD sickness absence, we defined three outcome variables: sickness absence (no = 0, yes = 1) at 3 months, 6 months, and 12 months after reporting sick with CMDs.

### Predictor Variables

The predictor variables age (≤50 years = 0; >50 years = 1), educational level (low i.e., primary education and junior secondary vocational or general education = 0; high i.e. senior secondary vocational or general education, higher professional education, and university = 1) and recovery expectations (≤3 months = 0; >3 months = 1) were defined according to the development study [9].

Depressive and anxiety symptoms were measured with the Four-Dimensional Symptom Questionnaire (4DSQ), which has shown good psychometric properties in the working population [13, 14]. All 4DSQ items were scored on a 5-point response scale using categories ‘no’(=0), ‘sometimes’ (=1), ‘regularly’ (=2), ‘often’ (=2), and ‘very often’ (=2). The depression scale consists of 6 items (Cronbach’s α = 0.91) with a score range 0–12; scores ≤2 were interpreted as absence of depressive symptoms and scores >2 as presence of depressive symptoms [14]. The anxiety scale consists of 12 items (α = 0.89) with a score range 0–24; scores ≤8 represented absence and scores >8 presence of anxiety symptoms [14]. Dichotomized (i.e., absent = 0, present = 1) depressive and anxiety scores were summed; a sum score = 0 was interpreted as absence of depressive and anxiety symptoms and scores 1 and 2 as presence of depressive and/or anxiety symptoms.

### Work-Related Factors

Psychosocial work characteristics were measured with the Questionnaire on the Experience and Evaluation of Work (QEEW) [15, 16]. We used the QEEW scales quantitative demands (11 items; α = 0.92), emotional demands (7 items, α = 0.78), ‘variety in work’ (6 items, α = 0.88), autonomy in work (11 items, α = 0.91), control over work (8 items, α = 0.88), and support from co-workers (9 items, α = 0.83) and supervisor (10 items, α = 0.93). Responses on these scales were rated on a four-point frequency scale ranging from “never” (=0) to “always” (=3). The sum scores of each scale were standardized as percentage of the maximum scale score, so that scores ranged from 0 to 100. In the analyses, each work-related factor was included as continuous variable.

Work–family conflict was investigated with the Work–Family Interface Scale [17]. Negative work-to-family (α = 0.82) and negative family-to-work (α = 0.72) spillover were measured by 3 items each with five-point frequency responses ranging from “never” (=0) to “very often” (=4); scores were summed (range 0–12) so that higher sum scores reflecting a more conflicting work–family interface. Work-to-family and family-to-work spillover were each included in the analyses as continuous variable.

### External Validation of the Prediction Rule

External validation of the prediction rule was done with IBM SPSS Statistics for Windows, version 20 (IBM Corp. Armonk, NY, released 2011). Using the logistic regression coefficients from the study sample of Nieuwenhuijsen et al. [9] we composed three linear predictors (LPs):

- LP1 = 0.129 − (0.619 * age) − (0.692 * education) − (1.080 * expected recovery) − (0.949 * symptoms) for the risk of being absent at 3 months,
- LP2 = 1.436 − (0.760 * age) − (1.047 * education) – (0.936 * expected recovery) – (0.860 * symptoms)for the risk of being absent at 6 months, and
- LP3 = 2.719 − (1.044 * age) − (1.154 * education) − (0.953 * expected recovery) − (0.552 * symptoms) for the risk of being absent at 12 months.

Mean predicted risks were plotted against observed frequencies of CMD sickness absence in a calibration graph. Calibration (i.e., the accuracy of predicted risks) is perfect if calibration graph intercept = 0 and slope = 1. In this study, we considered calibration adequate for non-significant (i.e., *P* ≥ 0.05) tests for calibration intercept and slope; miscalibration was concluded for *P* < 0.05 [18].

Discrimination between employees with and without CMD sickness absence 3, 6, and 12 months after reporting sick with CMDs was examined with ROC-curves. The area under the ROC-curve (AUC) is a measure for discrimination. AUC > 0.90 reflects perfect, 0.80–0.89 good, 0.70–0.79 fair, and 0.60–0.69 poor discrimination; AUC = 0.50 reflects no discrimination above chance.

### Updating of the Prediction Rule

Until now, we kept the logistic regression coefficients fixed at their original value obtained from the study sample of Nieuwenhuijsen et al. [9]. The prediction rule was re-calibrated by estimating logistic regression coefficients based on the data of the present study population [18]. Then, we added each work variable separately to the re-calibrated prediction rule. Improvement of the prediction rule’s ability to discriminate between employees with and without CMD sickness absence was investigated with Integrated Discrimination Improvement (IDI) [19, 20]. IDI = 0 represents no discrimination improvement after adding the work variable; IDI > 0 reflects significant discrimination improvement and IDI < 0 significant worsening of risk discrimination. IDIs were calculated in R (Project for Statistical Computing) using the predictABEL package [21].

## Results

A total of N = 596 employees reported sick with CMD in the period September 2013 to April 2014, of whom N = 220 (37 %) agreed to participate in the study. Participants were OP-diagnosed with emotional disturbances (N = 31), mood disorders (N = 22), and neurotic disorders (N = 164); three participants were excluded because they were OP-diagnosed within other ICD-10 F-categories. The questionnaire data of another 6 employees could not be linked to the occupational health service register. Consequently, 211 (35 %) participants with complete data were included in the analyses (Table 1). The majority had a high educational level and worked as administrator (20 %), manager (10 %), healthcare professional (10 %), consultant (10 %), project leader and supervisor (8 %), or teacher (7 %).

**Table 1:** Sample characteristics (N = 211)

	Mean (SD^a^)	n (%)
Age in years	44.1 (11.9)	
≤50 years		140 (67)
>50 years		68 (32)
Missing		3 (1)
Gender		
Men		90 (43)
Women		99 (47)
Missing		22 (10)
Educational level		
Low		14 (7)
High		190 (90)
Missing		7 (3)
Recovery expectations in months	3.2 (2.7)	
≤3 months		139 (66)
>3 months		50 (24)
Missing		22 (10)
Depression (range 0–12)	3.5 (3.4)	
Anxiety (range 0–24)	6.4 (5.8)	
Symptoms		
No		79 (37)
Yes		111 (53)
Missing		21 (10)
Work factors (range 0–100)		
Quantitative demands	55.3 (19.7)	
Emotional demands	33.7 (17.3)	
Variety in work	56.2 (22.2)	
Autonomy in work	47.4 (19.2)	
Control over work	37.3 (19.5)	
Co-worker support	68.4 (15.7)	
Supervisor support	64.5 (21.5)	
Work–home interference (range 3–12)		
Work to family spillover	6.8 (2.2)	
Family to work spillover	4.7 (1.7)	

^a^Standard deviation

### External Validation of the Prediction Rule

The participants had a median CMD sickness absence duration of 155 (interquartile range [IQR] 98–244) days. Three months after reporting sick, N = 122 (58 %) participants were still absent from work. Tests of calibration intercept and slope were significant, indicating that the original rule did not accurately predict the risk of being absent 3 months after reporting sick with CMDs (Table 2). Table 2 shows that discrimination between employees with and without CMD sickness absence at 3 months was poor.

**Table 2:** External validation of the prediction rule in 211 employees

Sickness absent at	N (%)	Calibration		Discrimination
		Intercept (SE^a^)	Slope (SE^a^)	AUC (95 % CI)^b^
3 months	122 (79)	1.94 (0.55)	0.31 (0.31)	0.54 (0.41; 0.65)
6 months	73 (47)	0.42 (0.18)	0.06 (0.23)	0.50 (0.40; 0.61)
12 months	18 (12)	n.a.	n.a.	n.a.

*n.a.* Not analyzed because of small sample size

^a^Standard error

^b^Area under the receiver operating characteristic curve (95 % confidence interval)

Six months after reporting sick with CMD, N = 73 (35 %) participants were still absent from work. The prediction rule did not accurately predict the risk of CMD sickness absence and failed to discriminate between employees with and without CMD sickness absence at 6 months (Table 2). Eighteen (9 %) participants were still absent from work 12 months after reporting sick with CMD. This number was too small to validate the prediction rule for CMD sickness absence at 12 months.

### Update of the Prediction Rule

When the prediction rules were re-calibrated based on study population data, discrimination improved for CMD sickness absence at 3 months (AUC = 0.69; 95 % CI 0.59–0.80), but not for CMD sickness absence at 6 months (AUC = 0.55; 95 % CI 0.45–0.65). When the work-related factor ‘variety in work’ was added to the prediction rule, discrimination improved to AUCs of 0.74 (95 % CI 0.63–0.85) and 0.62 (95 % CI 0.52–0.72) for CMD sickness absence at 3 and 6 months, respectively. The other work-related factors did not significantly improve discrimination (Table 3).

**Table 3:** Update of the prediction rule with work-related factors

Work-related factor	3 months	6 months
	IDI (95 % CI)^a^	IDI (95 % CI)^a^
Quantitative demands	1.27 (–0.52; 3.07)	2.33 (–0.00; 5.00)
Emotional demands	0.00 (–1.01; 0.90)	1.45 (–0.01; 3.55)
Variety in work	4.71 (0.61; 8.81)*	2.93 (0.00; 5.77)*
Autonomy in work	0.52 (–0.84; 1.88)	0.44 (–0.43; 0.13)
Control over work	1.02 (–0.65; 1.68)	1.00 (–0.64; 2.64)
Co-worker support	–0.25 (–0.96; 0.46)	0.57 (–0.27; 1.42)
Supervisor support	0.65 (–0.00; 1.38)	1.22 (–0.00; 2.81)
Work to family spillover	0.72 (–0.35; 1.78)	0.00 (–0.35; 0.49)
Family to work spillover	0.68 (–0.90; 2.26)	0.27 (–0.31; 0.85)

* Indicates discrimination improvement significant at the 5 % level

^a^Integrated Discrimination Improvement (95 % confidence interval)

Table 4 shows the added value of ‘variety in work’ differentiated by the items included in the scale. The items ‘repetitious work’ and ‘task variety’ improved the discriminative ability of the prediction rule for CMD sickness absence at 3 months. The scale item ‘varied work’ improved the discriminative ability of the prediction rule for CMD sickness absence at 6 months.

**Table 4:** Update the prediction rule with variety in work items

Variety in work items	3 months	6 months
	IDI (95 % CI)^a^	IDI (95 % CI)^a^
Do you repeatedly have to do the same things in your work?	8.27 (2.76; 13.76)*	0.41 (–0.50; 1.33)
Does your work require creativity?	2.93 (–0.59; 6.35)	0.19 (–0.22; 0.59)
Is your work varied?	2.11 (–0.66; 4.89)	2.97 (0.00; 5.90)*
Does your work require personal input?	1.76 (–0.69; 4.21)	2.60 (–0.00; 5.23)
Does your work sufficiently require all your skills and capacities?	2.07 (–0.49; 4.63)	2.19 (–0.28; 4.65)
Do you have enough variety in your work?	3.87 (0.17; 7.56)*	0.64 (–0.56; 1.84)

* Indicates discrimination improvement significant at the 5 % level

^a^Integrated Discrimination Improvement (95 % confidence interval)

## Discussion

The original prediction rule for identifying employees at risk of long duration CMD sickness absence was externally validated in a heterogeneous working population. The results showed miscalibration (i.e., the prediction rule did not accurately predict the risk of CMD sickness absence durations of 3 and 6 months) and poor discrimination between employees with and without CMD sickness absence 3 and 6 months after reporting sick with CMDs. Discrimination improved when the prediction rule was re-calibrated to the present study population and when ‘variety in work’ was added to the prediction rule. The other psychosocial work characteristics and work-family conflicts did not improve discrimination.

Previously, Nieuwenhuijsen et al. (2006) reported AUCs of 0.68 and 0.71 for sickness absence at 3 and 6 months after reporting sick with CMDs. A potential reason for finding poorer discrimination in the present study is that prediction rules generally perform better in development than in validation samples [18]. This phenomenon, known as over-optimism, can be problematic when prediction rules are fitted to the data of relatively small development samples. Nieuwenhuijsen et al. [9] corrected for over-optimism by internal validation, fitting the prediction rule to the original data as well as to each of 1000 bootstrap samples. Bootstrapping is a powerful approach to correct for over-optimism and, therefore, it is not likely that the poorer discrimination found in the present study can be explained by over-optimistic performance of the prediction rule in the development study.

The poor discrimination found in the present study might be due to differences between the study populations. When validating a prediction model, researchers should consider the relatedness between the development and validation samples [22]. The development sample comprised employees (40 % men and 30 % aged >50 years) with diverse occupations, but teachers constituted a relatively large proportion (54 %) of the sample.

Our current study population (42 % men and 32 % aged >50 years) only included 7 % teachers and may therefore differ too much from the development sample. When re-calibrated to the data of the present study population, discrimination by the prediction rule for sickness absence 3 months after reporting sick with CMDs was similar to that reported by Nieuwenhuijsen et al. [9].

An alternative explanation for the different results might be sought in how CMDs were diagnosed. In the development study, Nieuwenhuijsen et al. performed a Composite International Diagnostic Interview (CIDI), diagnosing 36 % of the employees with depressive or anxiety disorders. In the present study, 58 % of the participants presented with depressive or anxiety symptoms as measured with the 4DSQ. This difference could be indicative of diagnostic misclassification or represent a real case-mix difference in the sense that our study included more severe CMDs. Age, gender, OP-diagnoses, and CMD sickness absence duration of study participants were compared with age, gender, OP-diagnoses, and CMD sickness absence duration in 7909 employees of all (i.e., with and without sickness absence insurance) companies, who reported sick with CMDs in the baseline period. The latter were younger (mean age 41.0 years; *t* test *P* < 0.01) than the study participants, but did not differ in gender distribution (47 % women; Chi square *P* = 0.77). They were less often OP-diagnosed with neurotic disorders (62 %, Chi square *P* = 0.02) and had shorter CMD sickness absence duration (median 107 days, Mann–Whitney *P* < 0.01) than the study participants, which indicates that the present study might have included participants with more severe CMDs.

Another explanation for the poor performance of the prediction rule could be sought in the different time frames. The current study was conducted 9 years after developing the prediction rule. Meanwhile, the treatment and management of CMDs has changed. Therapy now advocates to add work-directed interventions to the treatment of CMDs [23–25]. This may have changed attitudes towards work and recovery expectations of employees with CMDs. In addition, economic and labor market changes in the past 9 years may have affected CMD sickness absence durations, attributing to our different findings.

### Study Strengths and Limitations

The prospective design of the study and the use of registered sickness absence data are assets of the study. A further advantage is that our study included a heterogeneous working population, although the 37 % participation rate restricts the generalizability of results to other populations of employees sick-listed with CMDs. When comparing the study participants with all employees who reported sick with CMDs in the same time frame, we found that our study might have included employees suffering more severe CMDs with longer median sickness absence durations. In addition, companies which have sickness absence insurances are generally small companies staffing up to 100 employees.

Work-related factors were studied by self-administered online questions. Although by far the most widely used way to assess psychosocial work environment characteristics, self-reported measures might be influenced by personal dispositions, mood, expectations, previous experiences, and health [26, 27]. Hence, differential and non-differential misclassification could not be excluded and might explain why work-related factors did not improve discrimination between worker with and without sickness absence at 3 and 6 months after reporting sick with CMDs. The finding that work-related factors do not improve discrimination between employees with and without long duration sickness absence is in line with results from previous studies on predictions of high sickness absence days in Norwegian nurses and Danish eldercare workers [28, 29]. This indicates that, despite being associated with sickness absence duration, psychosocial work characteristics do not discriminate between employees with and without long-term duration sickness absence.

### Practical Implications and Directions for Further Research

The original prediction rule poorly discriminated between employees with and without sickness absence at 3 and 6 months after reporting sick with CMDs. Discrimination improved when the prediction rule was re-calibrated to the data of present study population. When ‘variety in work’ was added, the re-calibrated prediction rule discriminated between employees with and without sickness absence at 3 months with AUC = 0.74. In other words, if we randomly select an employee who is still absent from work at 3 months and an employee who has fully resumed work at 3 months, the prediction rule will correctly assign the highest risk to the employee who is still absent from work in 74 % of the cases. However, re-calibrating the model for the present study population and adding additional variables creates a new prediction rule. Therefore, we still have to test the discriminative performance of the prediction rule in other validation studies of employees sick-listed with CMDs.

For practical use, predictor variables have to be readily available or easy to obtain by healthcare providers. The scale measuring ‘variety in work’ consists of 6 items, which could be administered during consultations with employees. If further research shows that the prediction rule with ‘variety in work’ applies to untested settings, then health care providers could use the prediction rule in first consultations with employees sick-listed with CMDs to identify those at risk of long (i.e., >3 months) duration CMD sickness absence. This tertiary preventive approach would enable health care providers to decide in an early stage of sickness absence to refer employees to interventions aimed at recovery and return to work [23, 24]. To facilitate its use in occupational healthcare practice, the prediction rule has to be modified into a simpler format and cut-off points have to be determined to decide which employees to refer. Furthermore, it remains to be investigated whether using the prediction rule to early refer high-risk employees to interventions facilitates return to work and improves the return to work prognosis of CMD sickness absence.

## Conclusion

The original prediction rule poorly discriminated between employees with and without CMD sickness absence at 3 and 6 months after reporting sick. When ‘variety in work’ was added as predictor variable, the prediction rule became a potential tertiary preventive tool to identify employees at risk of long-term (i.e., >3 months) CMD sickness absence and refer them to interventions aimed at recovery and return to work in an early stage of CMD sickness absence.
